# X-ray scattering study of GaN nanowires grown on Ti/Al_2_O_3_ by molecular beam epitaxy

**DOI:** 10.1107/S1600576723001486

**Published:** 2023-03-09

**Authors:** Vladimir M. Kaganer, Oleg V. Konovalov, Gabriele Calabrese, David van Treeck, Albert Kwasniewski, Carsten Richter, Sergio Fernández-Garrido, Oliver Brandt

**Affiliations:** a Paul-Drude-Institut für Festkörperelektronik, Leibniz-Institut im Forschungsverbund Berlin e.V., Hausvogteiplatz 5-7, 10117 Berlin, Germany; b ESRF – The European Synchrotron, 71 avenue des Martyrs, 38043 Grenoble, France; c Istituto per la Microelettronica e Microsistemi, Consiglio Nazionale delle Ricerche, via Gobetti 101, 40129 Bologna, Italy; d Leibniz-Institut für Kristallzüchtung (IKZ), Max-Born-Strasse 2, 12489 Berlin, Germany; e Institute for Optoelectronic Systems and Microtechnology (ISOM) and Materials Science Department, Universidad Politécnica de Madrid, Avenida Complutense 30, 28040 Madrid, Spain; Montanuniversität Leoben, Austria

**Keywords:** GaN nanowires, grazing-incidence small-angle X-ray scattering, GISAXS, molecular beam epitaxy, topotaxy

## Abstract

GaN nanowires (NWs) grown by molecular beam epitaxy on Ti films sputtered on Al_2_O_3_ are studied by X-ray diffraction (XRD) and grazing-incidence small-angle X-ray scattering (GISAXS). XRD, performed both in symmetric Bragg reflection mode and at grazing incidence, reveals Ti, TiN, Ti_3_O, Ti_3_Al and Ga_2_O_3_ crystallites with in-plane and out-of-plane lattice parameters intermediate between those of Al_2_O_3_ and GaN. These topotaxial crystallites in the Ti film, formed as a result of interfacial reactions and N exposure, possess little misorientation with respect to Al_2_O_3_. As a result, GaN NWs grow on the top TiN layer, possessing a high degree of epitaxial orientation with respect to the substrate. The measured GISAXS intensity distributions are modelled by the Monte Carlo method, taking into account the orientational distributions of NWs, the variety of their cross-sectional shapes and sizes, and the roughness of their side facets. The cross-sectional size distributions of the NWs and the relative fractions of the {1100} and {1120} side facets are determined.

## Abstract 2

Monte Carlo modelling of grazing-incidence small-angle X-ray scattering intensity provides detailed information on the cross-sectional size and shape distributions of GaN nanowires, as well as the roughness of their side facets. The cross sections are found to be dodecagons rather than hexagons. A narrow orientational distribution of the nanowires is found using X-ray diffraction. In addition, a variety of different topotaxial crystallites with in-plane and out-of-plane lattice parameters intermediate between those of Al_2_O_3_ and GaN are revealed in the sputtered Ti film.

## 1. Introduction

Semiconductor nanowires (NWs) have essential advantages over epitaxial films of the same materials due to the large areas of their side facets and the capability for free elastic relaxation of the material, which provides both a reduction in the density of lattice defects near the interface to the substrate and defect-free interfaces in axial and radial NW heterostructures. The self-induced growth of GaN NWs (Fernández-Garrido *et al.*, 2009 ▸; Geelhaar *et al.*, 2011 ▸), in contrast to the vapour–liquid–solid (VLS) growth of the majority of semiconductor materials, does not involve metal particles at the top (Ristić *et al.*, 2008 ▸). GaN NWs grow on various substrates in dense arrays and, as a consequence of the self-induced growth, their density cannot easily be controlled by varying the temperature or atomic fluxes. NWs in dense arrays shadow the side facets of each other from the impinging fluxes (Sibirev *et al.*, 2012 ▸; Sabelfeld *et al.*, 2013 ▸), hindering the growth of radial heterostructures, and they also bundle together (Kaganer *et al.*, 2016 ▸). TiN is a substrate with a low nucleation rate of GaN NWs, resulting in a density an order of magnitude lower than that of GaN NWs grown on Si(111). TiN layers have been prepared by nitridation of Ti films (Sarwar *et al.*, 2015 ▸; Wölz *et al.*, 2015 ▸; Zhao *et al.*, 2016 ▸; van Treeck *et al.*, 2018 ▸; Mudiyanselage *et al.*, 2020 ▸) and Ti foils (Calabrese *et al.*, 2016 ▸; May *et al.*, 2016 ▸; Calabrese *et al.*, 2017 ▸; Ramesh *et al.*, 2019 ▸, 2020 ▸; Mudiyanselage *et al.*, 2020 ▸), as well as by directly sputtering TiN_*x*_ on Al_2_O_3_ (Auzelle *et al.*, 2021 ▸).

X-ray scattering methods are widely used to study semiconductor NWs. In most cases, NWs are faceted and have the cross-sectional shape of a hexagonal prism. The scattered intensity has maxima in the directions of the normals to the facets. These facet truncation rods in reciprocal space correspond to a hexagon in real space and are similar to the crystal truncation rods in diffraction from planar surfaces (Robinson, 1986 ▸). Characteristic six-armed stars can be observed in the intensity patterns obtained by X-ray diffraction (XRD) of a focused X-ray beam on single GaAs NWs (Davtyan *et al.*, 2017 ▸; Al Hassan *et al.*, 2018 ▸). Arrays of group IV and III–V NWs grown by the VLS technique possess narrow orientational distributions. As a result, facet truncation rods are observed in XRD patterns from arrays of GaAs (Mariager *et al.*, 2007 ▸; Schroth *et al.*, 2018 ▸), Si (David *et al.*, 2008 ▸; Buttard *et al.*, 2013 ▸) and InAs (Eymery *et al.*, 2007 ▸, 2009 ▸; Mariager *et al.*, 2009 ▸) NWs. These maxima in the directions of the facet normals are also present in the grazing-incidence small-angle X-ray scattering (GISAXS) intensity from Si NWs (David *et al.*, 2008 ▸).

In contrast to the VLS-grown NWs of other III–V compounds, the self-induced growth of GaN NWs gives rise to broad distributions of orientations of individual NWs in the array. This concerns both the orientation of the long axes (tilt) and the in-plane orientation of the crystal lattice and the side facets (twist) (Horák *et al.*, 2008 ▸; Jenichen *et al.*, 2011 ▸; Geelhaar *et al.*, 2011 ▸). We note here that GaN NWs are free of dislocations along their axes, and the Eshelby twist (Eshelby, 1953 ▸) in individual NWs is absent. Recently, we have applied GISAXS to study dense arrays of GaN NWs on Si(111) (Kaganer *et al.*, 2021 ▸). We found that the facet truncation effect is partially blurred because of the misorientation between individual NWs in the array.

The X-ray intensity distribution has been modelled by a Monte Carlo method that takes into account the distributions of the NW lengths, the cross-sectional sizes and shapes and the orientations. GISAXS provides statistical information on the average radius and the width of the radius distribution of a NW array. It is also sensitive to the roughness of the side facets of NWs, which is less than 1 nm, *i.e.* three to four times the height of the atomic steps. The approach was developed initially for NWs represented by prisms with hexagonal cross sections (Kaganer *et al.*, 2021 ▸), *i.e.* with the GaN{ 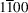 } side facets, and then also applied to NWs with both { 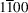 } and { 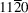 } side facets to determine the ratio of the areas of these two facets (Volkov *et al.*, 2022 ▸).

In the present paper, we apply GISAXS to study GaN NWs on nitridated Ti films sputtered on Al_2_O_3_(0001). We develop further the approach proposed for the analysis of dense arrays of GaN NWs on Si(111). The GISAXS intensity distribution contains a weaker intensity from a lower NW density which overlaps with a stronger parasitic signal from the sputtered film. This makes the analysis more complicated. These two contributions can be distinguished in the intensity pattern and the NW intensity can be extracted, since NWs are needle-shaped oriented objects whose intensity in reciprocal space resembles a disc perpendicular to the long axis of the NWs. By comparing the measured and Monte Carlo simulated intensities, we find the distribution of the NW radii and that of the ratios of the { 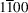 } and { 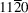 } side facets, as well as the roughnesses of these facets.

The Monte Carlo modelling of the GISAXS intensity requires as an input the range of orientations of the long axes (tilt) and that of the side facets (twist) of the NWs in the ensemble. As a prerequisite for the GISAXS study, we performed XRD measurements with the primary purpose of determining these ranges from the widths of the respective reflections on sample rotation. We found that the arrays of GaN NWs on Ti/Al_2_O_3_ possess notably smaller tilt and twist ranges than their counterparts on Si(111).

The samples studied in the present paper were cut from the same wafers as the samples studied by Calabrese *et al.* (2020 ▸). While the former work was devoted to an analysis of strain in the NWs and respective precise measurements of the positions of the GaN XRD peaks, the focus of the present paper is a GISAXS study of the NW sizes and shapes. We used XRD to obtain the ranges of misorientation of NWs in the array, both normal to the substrate surface and parallel to it (tilt and twist), and did not analyse the precise positions of the XRD peaks. The XRD measurements also revealed crystalline phases formed in the sputtered Ti film as a result of chemical reactions with the elements in the substrate and the NWs, shedding light on the surprisingly narrow orientational distributions of the NWs. Hence, we present below the results of XRD in some detail, prior to presenting the GISAXS results.

## 2. Experimental details

As already mentioned in the *Introduction* [Sec sec1], the samples studied in the present work were cut from the same wafers as the samples studied by Calabrese *et al.* (2020 ▸). We keep the same notation A–D for the samples and refer to the former paper for the nominal growth conditions. The samples were grown by plasma-assisted molecular beam epitaxy (PA-MBE) on Ti films sputtered on Al_2_O_3_(0001). Before NW growth, a Ti film with a thickness of either 1.3 µm (sample A) or 3.4 µm (samples B–D) was deposited on bare Al_2_O_3_(0001) by magnetron sputtering, as described by van Treeck *et al.* (2018 ▸). After Ti sputtering, the samples were loaded into the growth chamber of the PA-MBE system, being exposed to air during the transfer. The MBE system is equipped with a solid-source effusion cell for Ga and a radio-frequency N_2_ plasma source for active N. For samples B and C, a dedicated nitridation step was introduced before NW growth. The N flux used for substrate nitridation was the same as that for GaN growth. After the intentional substrate nitridation process, the Ga shutter was opened to initiate the formation of GaN NWs. For samples A and D, the Ga and N shutters were opened simultaneously. The XRD measurements presented in Section 3.1 show that the final content of the sputtered film was the same as for samples B and C, so that the same nitridation process took place during NW growth. Further details concerning the nitridation process are described by Calabrese *et al.* (2019 ▸).

**Figure 1:**
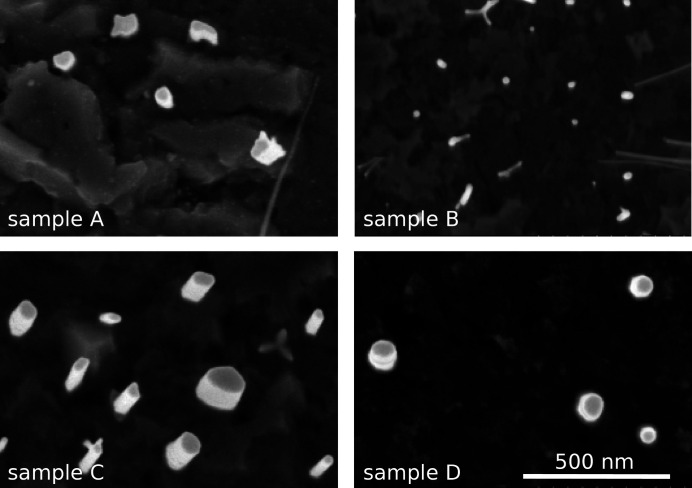
Top-view SE micrographs of samples A–D. The scale bar is common to all micrographs.

Fig. 1 ▸ presents top-view secondary electron (SE) micrographs of samples A–D. Similar micrographs were used by Calabrese *et al.* (2020 ▸) to obtain the average NW radii, as included in Table 1 ▸. However, the resolution of the SE micrographs is not good enough to reveal details of the cross-sectional shapes of the NWs, which we found through the GISAXS study below. A significant roughness of the substrate surface is also evident from the micrographs, giving rise to an additional contribution to the GISAXS intensity.

The laboratory XRD measurements were performed in two geometries, out-of-plane in a symmetric Bragg reflection mode and in-plane at grazing incidence. For the acquisition of out-of-plane reciprocal-space maps we used a Bruker D8 Discover diffractometer operating a Cu anode at 1.6 kW. The beam-conditioning optics comprised a Göbel mirror and an asymmetric two-bounce channel-cut Ge(220) monochromator. The beam was thus collimated to about 0.0085°. The diffracted intensity was recorded using a Mythen 1D position-sensitive detector with a channel pitch and width of 50 µm and 8 mm, respectively. Two-dimensional reciprocal-space maps were obtained by rocking scans of the sample.

The in-plane XRD measurements were performed using a Rigaku SmartLab high-resolution X-ray diffractometer in a horizontal scattering plane. The source was a 0.4 × 8 mm electron line focus on a rotating Cu anode operating at 9 kW. The beam was vertically collimated by a Göbel mirror, and an asymmetrically cut two-bounce Ge(220) monochromator was used to select the Cu *K*α_1_ emission line. The beam size was reduced to 0.3 × 5 mm by slits and the angular resolution was defined by horizontal Soller slits of 0.25 and 0.228° angular acceptance for the incident and scattered beams, respectively. The incidence angle was set to 0.25°. Diffraction intensities were recorded using a Hypix-3000 area detector with 100 × 100 µm pixel size.

Grazing-incidence X-ray diffraction (GID) reciprocal-space maps were acquired on beamline ID10 at the European Synchrotron Radiation Facility (ESRF, Grenoble, France) at an X-ray energy of 22 keV (wavelength λ = 0.5636 Å). A linear detector (Mythen 1K, Dectris) was placed parallel to the substrate surface to cover the range of scattering angles around the diffraction angle of the GaN( 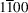 ) reflection with 2θ ≃ 11.7°. The grazing-incidence angle was 0.12° and the exit angle was 0.24°. The sample was rotated about the substrate surface normal and linear detector scans were recorded for different azimuthal angles ω. The obtained reciprocal-space maps are similar to the ω–2θ maps measured in laboratory XRD experiments. We refer to them as ω–2θ maps, although the sample rotation axis was along the surface normal, in contrast to symmetric Bragg reflections in the laboratory XRD measurements where the sample rotation axis lay in the surface plane.

GISAXS measurements were also performed on beamline ID10 at the ESRF at the same X-ray energy of 22 keV. The incident beam was directed at grazing incidence to the substrate. The grazing angle was chosen for each sample from several trials to provide the best signal from the NWs. It exceeds the critical angle of total external reflection for Al_2_O_3_ (0.1° at the used energy) by at least 2.5 times, allowing us to avoid possible complications of the scattering pattern typical for grazing-incidence X-ray scattering (Renaud *et al.*, 2009 ▸). The X-ray beam incident on the sample was focused by beryllium compound refractive lenses to a size at the sample position of 135 µm laterally and 13 µm vertically (in the direction of the surface normal). At an incidence angle of 0.25°, the size of the spot illuminated by the incident beam at the sample was 3 × 0.135 mm. Thus, with a typical NW density of 1 × 10^9^ cm^−2^, approximately 4 × 10^6^ NWs were illuminated simultaneously. A two-dimensional detector (Pilatus 300K, Dectris) was placed at a distance of 2.38 m from the sample. The angular width of a detector pixel was 8.06 × 10^−3^ nm^−1^.

## 3. Results

### 3.1. XRD

**Figure 2:**
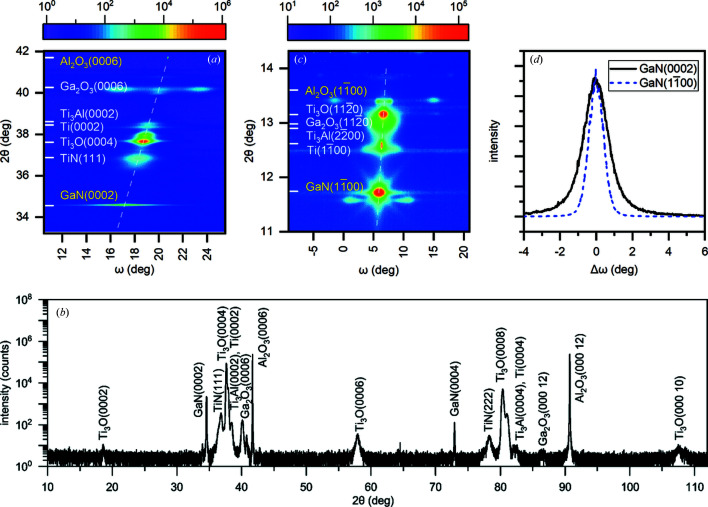
(*a*) An XRD reciprocal-space map in the vicinity of the GaN(0002) reflection, (*b*) a θ–2θ scan over a wide angular range, (*c*) a GID reciprocal-space map in the vicinity of the GaN( 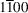 ) reflection and (*d*) intensity profiles through the GaN reflections from sample C. The colour-coded scale bars represent the intensity on the maps in counts, while the dashed lines show the θ–2θ radial scans across the Al_2_O_3_ substrate and the GaN NW reflections. The peaks along these lines in between the substrate and the NW reflections are due to different crystalline phases emerging because of interfacial reactions in the sputtered Ti film on the substrate. The reflections of various phases are indexed.

Laboratory XRD measurements of the ω–2θ reciprocal-space maps at the GaN(0002) reflection were performed to measure the range of the NW tilt. The maps for samples A–D are very similar. Fig. 2 ▸(*a*) presents such a map for sample C. One can see a very sharp Al_2_O_3_(0006) reflection and a GaN(0002) reflection that is extended in the ω direction. The dashed line from the Al_2_O_3_(0006) to GaN(0002) reflections indicates the direction of a radial θ–2θ scan. There are a number of peaks in between, which are the result of the interfacial reactions in the sputtered Ti film (Selverian *et al.*, 1991 ▸; Li *et al.*, 1992 ▸; Koyama *et al.*, 1993 ▸; Kelkar & Carim, 1995 ▸; Calabrese *et al.*, 2019 ▸).

We indicate in Fig. 2 ▸(*a*) the 2θ angles for suitable compounds and reflections found in *Pearson’s Crystal Data* (Villars & Cenzual, 2010 ▸). We searched for hexagonal phases, with the basal plane parallel to the substrate surface, of compounds composed of Ti and the chemical elements of the substrate or the NWs. Since the positions of the diffraction peaks are affected by thermal strain, epitaxial strain and impurities in the crystals, we do not expect that the 2θ values will exactly coincide with the literature data. Also, there is a notable spread in the lattice parameters between the results of different studies collected in the database.

Fig. 2 ▸(*b*) shows a θ–2θ scan over a wide range of scattering angles. It is an extension of the intensity distribution along the dashed line in the map of Fig. 2 ▸(*a*) to both small and large angles. Higher-order reflections of the same phases that are identified in the map are revealed. Peaks of any other compound are not found. We note that high-resolution transmission electron microscopy shows that the top 40–80 nm of the Ti film is transformed to TiN by nitridation (Calabrese *et al.*, 2019 ▸). Hence, the GaN NWs grow on TiN.

Fig. 2 ▸(*c*) shows the GID ω–2θ map in the vicinity of the GaN( 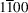 ) reflection of the same sample C. Upon rotation of the sample about the surface normal, the diffraction pattern of Fig. 2 ▸(*c*) is repeated after every 60° (Calabrese *et al.*, 2020 ▸). The dashed line represents the θ–2θ scan. The Al_2_O_3_( 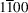 ) substrate reflection is not seen because the small incidence and exit angles prevent the penetration of the X-ray radiation into the substrate. This reflection is observed in a measurement with larger incidence and exit angles (not shown here). The scattering angles 2θ of the same phases as in Fig. 2 ▸(*a*) are indicated. We observe the in-plane reflections of the hexagonal crystals Ti, Ti_3_O, Ti_3_Al and Ga_2_O_3_. Cubic TiN does not have a Bragg reflection in the angular range of the map in Fig. 2 ▸(*c*).

Fig. 2 ▸(*d*) presents ω scans through the GaN(0002) and GaN( 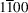 ) reflections, extracted from the maps for sample C. These curves are out-of-plane and in-plane orientation distributions (tilt and twist, respectively) in the NW ensemble. We recall here that a twist in individual NWs (Eshelby twist) is absent since the NWs do not contain dislocations, and the twist refers to the NW ensemble.

The FWHM of the NW tilt distribution is 1.52°. The strongest reflection in Fig. 2 ▸(*a*) is Ti_3_O(0004). It possesses a width in the ω direction of 0.5°, *i.e.* the tilt of the Ti_3_O crystallites is one-third that of the GaN NWs. Similar measurements of the NW tilt distributions on the other samples give close results, summarized in Table 1 ▸.

The FWHM of the GaN( 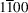 ) reflection gives a twist in the NW array of sample C of 0.96°. For comparison, the measurement of the same reflection by triple-crystal diffraction on the laboratory diffractometer gives a somewhat smaller value of 0.89°. The difference can be explained by the geometric broadening in the synchrotron measurement with the linear detector. In the latter measurement, an ∼5 mm long stripe on the sample surface is illuminated by the incident X-ray beam and contributes to diffraction. Hence, the twist values obtained by the laboratory X-ray diffraction are more reliable and are given in Table 1 ▸.

### 3.2. GISAXS

**Figure 3:**
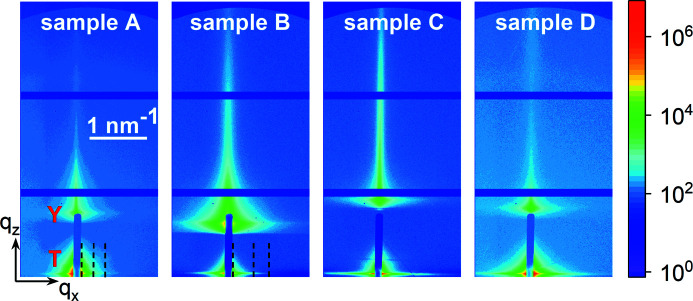
GISAXS intensity from samples A–D as measured by a two-dimensional detector. The vertical blue bar in the middle of each scattering pattern is the beamstop. The scattering around the transmitted beam and the Yoneda peak are marked in the map of sample A by T and Y, respectively. The three vertical dashed lines in the maps of samples A and B mark the positions of the scans presented in Fig. 4. The colour-coded scale bar on the right, representing the intensity in counts, and the *q* scale bar in the map of sample A are applicable to all samples.

Fig. 3 ▸ presents the GISAXS intensity distributions for samples A–D. Each map comprises two peaks, the transmitted one and the Yoneda peak (Yoneda, 1963 ▸), labelled in the map of sample A as T and Y, respectively. The Yoneda peak is identified on the basis of its angular position on varying the incidence angle α_i_ (not shown): the angular distance between the transmitted peak and the Yoneda peak varies as α_i_ + α_c_, where α_c_ is the critical angle of total external reflection. The angular distance between the transmitted and reflected beams would vary as 2α_i_. However, the reflected beam is not seen, because of the large incidence angles and the significant roughness of the sputtered Ti layer (see Fig. 1 ▸).

Our analysis is focused on the small-angle scattering around the transmitted beam, at the bottom of each map in Fig. 3 ▸. The small-angle scattering intensity distribution consists of a halo around the direct beam direction and a horizontal (parallel to the substrate surface and normal to the long NW axis) streak. The halo stems from the scattering from the sputtered film on the substrate. The streak is due to scattering from the NWs, which are long rods that scatter in the plane perpendicular to the rods. The intersection of a disc in reciprocal space, corresponding to a rod in real space, with the detector plane gives rise to this streak.

**Figure 4:**
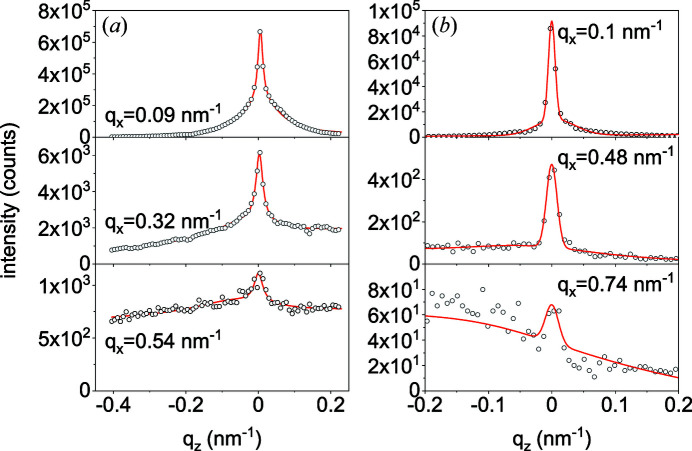
Measured intensity profiles (circles) along lines of constant *q*_*x*_ marked by the dashed lines in Fig. 3 ▸ and the respective fits (lines) for samples (*a*) A and (*b*) B. The measured intensity is fitted to the sum of two Gaussians, a narrow one representing the scattering from the NWs and a broader one due to scattering from the rough substrate. The background is assumed to depend linearly on *q*_*z*_.

The two contributions to the scattered intensity can be distinguished by analysing the scans in the surface normal direction. Some example scans for samples A and B are marked in Fig. 3 ▸ by dashed lines, and the intensity in these scans is shown in Fig. 4 ▸. The axes *q*_*x*_ and *q*_*z*_ are parallel to the substrate surface and normal to it, respectively (see Fig. 3 ▸). The scans are fitted to the sum of two Gaussians, a narrow one representing the X-ray scattering intensity from the NWs and a broad one due to the X-ray scattering from the sputtered film, plus a background that depends linearly on *q*_*z*_.

**Figure 5:**
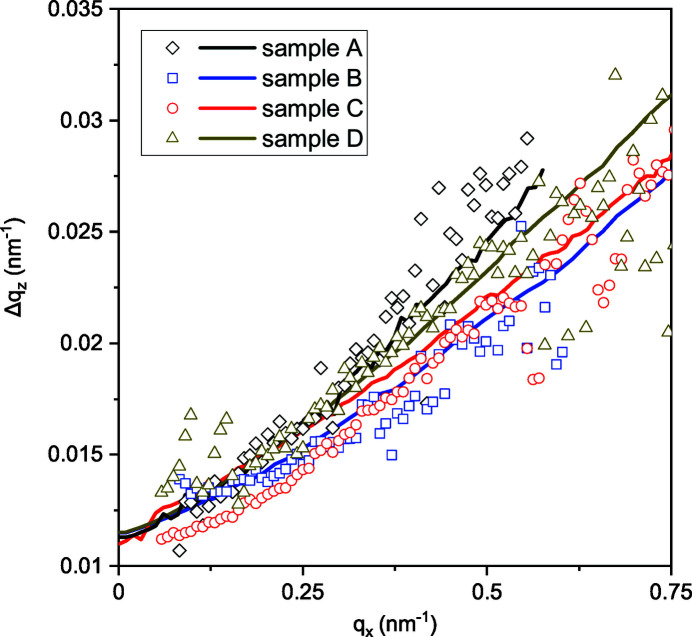
FWHMs of the intensity profiles Δ*q*_*z*_ as a function of the wavevector *q*_*x*_ (open symbols). Lines are the results of the Monte Carlo simulations, with the ranges of the NW tilt angles presented in Table 1 ▸ and a resolution of 1.2 × 10^−2^ nm^−1^.

Since each NW scatters in the plane perpendicular to its long axis, a range of orientations of the long axes (tilt) gives rise to a cone in the intensity distribution in reciprocal space and a fan in the intersection of this cone with the detector plane. Hence, in a zero-order approximation, the FWHM Δ*q*_*z*_ of a *q*_*z*_ scan of the intensity is expected to depend linearly on *q*_*x*_. The Δ*q*_*z*_(*q*_*x*_) dependencies obtained from the fits of the *q*_*z*_ scans for samples A–D are presented in Fig. 5 ▸. The dependencies are close to linear. The average slope Δ*q*_*z*_/*q*_*x*_ of the curves (translated to angular units) is about 1.7°, close to the tilt range in Table 1 ▸ measured by XRD. A more accurate evaluation of the Δ*q*_*z*_(*q*_*x*_) dependence is done in Section 3.3 by the Monte Carlo method. The finite intersection point *q*_*z*_ ≃ 1.2 × 10^−2^ nm^−1^ with the ordinate axis is also discussed in Section 3.3. It is mainly due to the finite detector resolution. The contribution due to finite NW lengths *L* (Δ*q*_*z*_ ∝ 2π/*L*) is smaller.

For further analysis, we need the GISAXS intensity *I*(*q*_*x*_) along the horizontal stripes in the reciprocal-space maps in Fig. 3 ▸. These stripes correspond to the maximum intensity of the *q*_*z*_ scans exemplified in Fig. 4 ▸, with the background scattering subtracted. We use a linear (as established above in the zero-order approximation) dependence of the widths Δ*q*_*z*_ on *q*_*x*_ in Fig. 5 ▸ to improve the fits of the *q*_*z*_ scans and extend the *q*_*x*_ range as much as possible to the regions of low intensity and substantial noise in the experimental data (see the bottom curves in Fig. 4 ▸). After a fit of the *q*_*z*_ scans from a reciprocal-space map has been performed and the widths Δ*q*_*z*_ obtained, the dependence Δ*q*_*z*_(*q*_*x*_), shown in Fig. 5 ▸, is fitted by a straight line. The fit of the *q*_*z*_ scans is then repeated, this time with the peak position and width thus predefined, rather than as free parameters of the fit. Since the only output of this second fit that we need is the maximum intensity, it can be determined at large *q*_*x*_, where the experimental data are noisy. The curves shown in Fig. 4 ▸ are the result of such two-step fits.

The small-angle scattering intensity *I*(*q*) from particles with a sharp change in the electron density at a surface is expected to follow Porod’s law, *I*(*q*) ∝ *q* ^−4^, at large *q* (Porod, 1951 ▸). The large-*q* limit is reached at *q* ≳ 2π/*R*, where *R* is a characteristic size of the particles, which is the NW radius in our case. Taking *R* = 20 nm for an estimate, we find that Porod’s law is expected to be applicable at *q* ≳ 0.3 nm^−1^, which is well inside the range of measured wavevectors in the maps of Fig. 3 ▸.

Porod’s law can be derived from Fresnel’s law for scattering from planar surfaces by an average over all orientations of the plane (Sinha *et al.*, 1988 ▸). In the present case of NWs epitaxially oriented with respect to the substrate, the average over orientations is incomplete because of preferential orientations of the side facets. This results in a dependence of the scattered intensity on the azimuthal orientation of the sample. A plot of the product 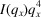 , rather than the intensity *I*(*q*_*x*_), highlights the deviation from Porod’s law and allows a more detailed analysis of the facet truncation-rod scattering (Kaganer *et al.*, 2021 ▸).

**Figure 6:**
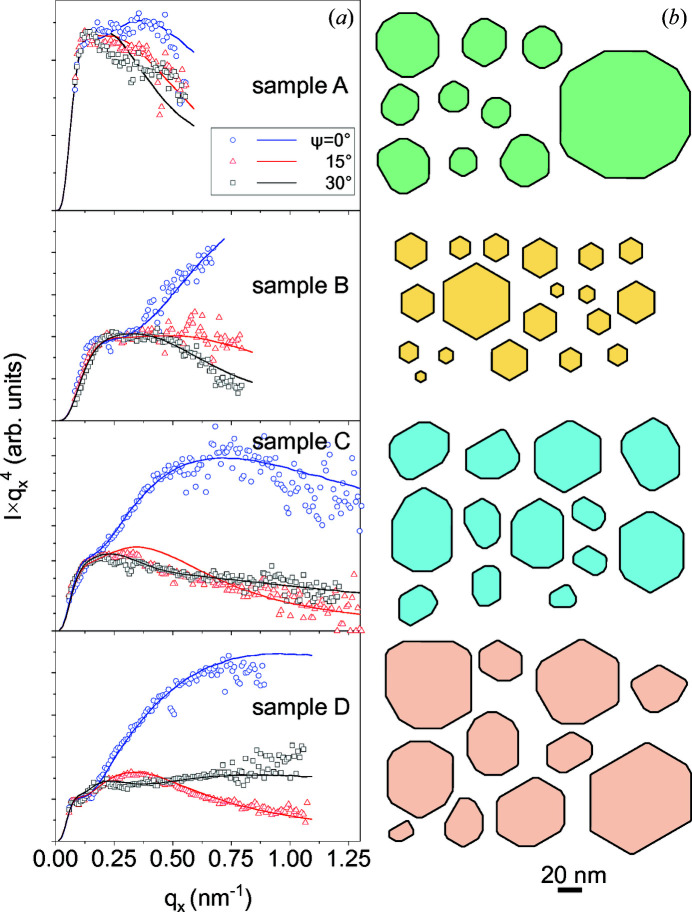
(*a*) GISAXS intensities *I*(*q*_*x*_) for samples A–D, presented as products 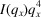 (open symbols) and the respective Monte Carlo simulations (lines). The measurements were performed for three different azimuthal orientations ψ of the incident X-ray beam with respect to the side facets of the NWs. (*b*) Examples of the cross sections of NWs used in the simulation of each sample. The scale bar is common for all samples.

Thus, we plot in Fig. 6 ▸ the GISAXS intensity for samples A–D as the product 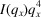 . The reciprocal-space maps, similar to those presented in Fig. 3 ▸, were measured with the azimuthal rotation of the samples about the vertical axis as an angle ψ from 0 to 90° with a step of 15°. The sixfold symmetry of the scattering patterns is established and the distinct intensity distributions obtained for ψ = 0, 15 and 30° are shown by circles, triangles and squares, respectively.

### 3.3. Monte Carlo simulations

We modelled the GISAXS intensity by the Monte Carlo method, as described by Kaganer *et al.* (2021 ▸). The NWs are considered as prisms with polygonal cross sections. The scattered intensity from a single NW can then be expressed through the coordinates of the vertices of the polygon. The simplest shape of the cross section is a regular hexagon limited by GaN{ 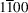 } facets. The hexagon sizes are assumed to obey a log-normal distribution in the Monte Carlo simulations. The calculated intensity curves in Fig. 6 ▸ were obtained by averaging the scattering intensity over about 5 × 10^5^ randomly generated NWs, a number that is an order of magnitude smaller than the number of NWs contributing to the measured GISAXS intensity, estimated in Section 2.

For samples A, C and D, regular hexagons are not sufficient to model the experimental data. Thus, we first randomly distort a hexagon while keeping the GaN{ 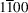 } side facets. Secondly, we introduce GaN{ 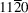 } planes in the NW shape. This is done by drawing lines whose orientation is the median of the orientations of the two adjoining sides of a hexagon. These lines cut out triangles at the vertices of the hexagon. The cross-sectional shape of the prism becomes a dodecagon (a polygon with twelve vertices) rather than a hexagon (a polygon with six vertices). Examples of such dodecagons used in the simulation of GISAXS intensity are presented in Fig. 6 ▸. The scattered intensity can still be calculated using the positions of the vertices. Log-normal distributions are assumed for the hexagon distortion and for the cuts of its apexes, with the parameters varied to fit the experimental curves. For each simulated polygon, we calculate its area *A* and perimeter *P*, as well as the parts of the perimeter 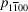 and 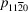 representing separately the { 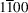 } and { 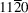 } facets, 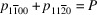 . The quantities of interest are the NW radius *R* = 2*A*/*P* and the fraction of { 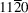 } facets, 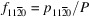 .

The NW array is simulated by randomly rotating the NWs about the horizontal and vertical axes in the angular ranges of the tilt and twist determined by the XRD measurements in Section 3.1 and presented in Table 1 ▸. We also take into account the roughnesses of the side facets of the NWs by including random shifts of the side facets in the direction of their normals by monolayer-height steps. A geometric distribution of steps is assumed and a roughness factor is calculated in the same way as is done in crystal truncation-rod calculations (Robinson, 1986 ▸). As a result, the contribution of each facet to the scattering amplitude contains an additional roughness factor (Kaganer *et al.*, 2021 ▸).

The lines in Fig. 5 ▸ are the result of a Monte Carlo simulation that takes into account the distributions of the NW cross-sectional sizes, lengths and orientations. We simulate the *q*_*z*_ dependence of the scattered intensity *I*(*q*_*z*_) at a given *q*_*x*_ and then fit this curve to a Gaussian, in the same way as was done with the experimental data. The *q*_*x*_ dependence of the FWHM Δ*q*_*z*_ of the Gaussians is close to a straight line, with the slope depending on the width of the distribution of the NW tilt angles. The FWHMs of the tilt angle distributions obtained by adjusting the simulated curves to the experimental ones are presented in Table 1 ▸. The values obtained in the Monte Carlo simulations of the GISAXS intensity are somewhat larger than those obtained from the XRD measurements.

The width Δ*q*_*z*_ at *q*_*x*_ = 0 has been treated by Kaganer *et al.* (2021 ▸) as a broadening solely due to the finite NW lengths *L*, Δ*q*_*z*_ ∝ *L* ^−1^. The lengths thus obtained were found to be smaller than the actual NW lengths and were attributed to the lengths of the segments of bundled NWs between the joints. In the present case of low NW density, the NWs are not bundled and their lengths, obtained from the side-view SE micrographs and given in Table 1 ▸, are used as an input in the Monte Carlo simulation. We find in the simulation that the finite-length broadening Δ*q*_*z*_ at *q*_*x*_ = 0 is notably smaller than the widths found in the experiment. We then take into consideration the finite resolution of the experimental curves and accordingly perform an average of the Monte Carlo intensities *I*(*q*_*z*_) over a range Δ*q* ^res^. The curves presented in Fig. 5 ▸ are obtained with the resolution Δ*q* ^res^ = 1.2 × 10^−2^ nm^−1^, which is 1.5 times the angular size of the detector pixel. This result can be considered as a partial exposure of the neighbouring detector pixels. Thus, the curves in Fig. 5 ▸ are obtained taking into account both the finite-length and the resolution broadening of the Monte Carlo simulated curves.

The lines in the plots in Fig. 6 ▸(*a*) are obtained by the Monte Carlo simulation, examples of the simulated cross-sectional shapes are shown in Fig. 6 ▸(*b*) and the parameters of the NW ensembles of the samples A–D are included in Table 1 ▸. Let us consider first the GISAXS intensity from sample B. Its dependence on the azimuth ψ is qualitatively similar to that of GaN NWs on Si(111) (Kaganer *et al.*, 2021 ▸). The product 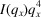 rises at large *q*_*x*_ at ψ = 0, decays at ψ = 30° and shows an intermediate behaviour at ψ = 15°. Such behaviour is expected for hexagonal cross sections and we find that the simulation of NWs by regular hexagons adequately describes the experiment. The maximum intensity at large *q*_*x*_ is the facet truncation-rod scattering and the minimum at ψ = 30° is the scattering in the direction of the angle between the facets. The rise in the 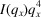 curve at small *q*_*x*_ is sensitive to the average NW radius, while a dip before a further rise of the curve at ψ = 0 provides the width of the radial distribution.

**Figure 7:**
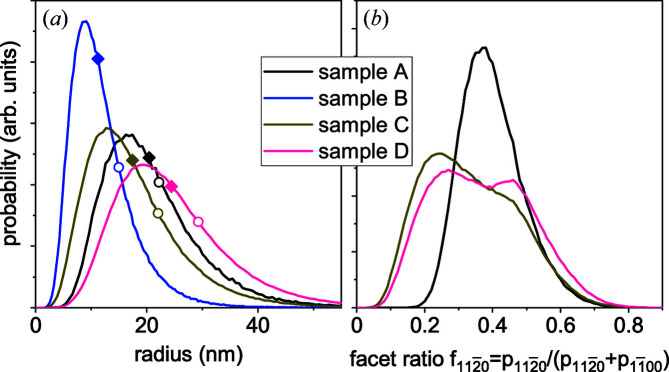
Probability distributions of (*a*) the radius and (*b*) the fraction of { 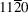 } facets obtained by the Monte Carlo simulations of the GISAXS intensity curves in Fig. 6 ▸. Filled diamonds on the curves in panel (*a*) show the average NW radii obtained in the present work, while open circles are the values obtained from SE micrographs by Calabrese *et al.* (2020 ▸).

The distribution of the NW radii obtained in the Monte Carlo simulation is shown in Fig. 7 ▸(*a*). We find a mean radius of 11.2 nm and a standard deviation of the radial distribution of 5.2 nm. Sample B exhibits the thinnest NWs from the series under investigation. The mean radii for this and other samples are marked in the plot by filled diamonds. We also mark, by open circles on the respective curves in Fig. 7 ▸(*a*), the mean radii obtained by Calabrese *et al.* (2020 ▸) from top-view SE micrographs and reproduced in Table 1 ▸. The mean radii thus obtained are systematically larger by 2 to 5 nm than those obtained from GISAXS in the present work. The difference can be attributed partially to the limited resolution of scanning electron microscopy and partially to the asymmetric radius distributions in Fig. 7 ▸(*a*), with the maximum value smaller than the average.

Fine-tuning of the Monte Carlo simulations to the experiment also requires that we include roughness with an r.m.s. value of σ = 0.7 nm. Since the height of a monolayer step at the side facet of an NW is equal to the GaN lattice parameter *a* = 0.319 nm, this value corresponds to only two atomic steps along the entire NW length.

Proceeding now to sample C, we find that the intensity curve at the intermediate azimuth ψ = 15° is not in between the curves for 0 and 30°, as is expected for oriented hexagons and observed for sample B. Rather, the curves at 15 and 30° almost coincide, and at large *q*_*x*_ the intensity at the azimuth of 15° is even smaller. Simulation of these intensity curves requires that we take into account both { 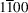 } and { 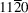 } side facets. Fig. 6 ▸ shows the result of the Monte Carlo simulation for sample C and examples of the cross sections used in the simulation. The NW radii are larger and the radius distribution is broader than for sample B [see Fig. 7 ▸(*a*)]. The distribution of the fraction 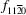 is shown in Fig. 7 ▸(*b*). A wide distribution of the ratios of different facets is needed to model the experimental curves. We note that for NWs on Si(111) the fraction of { 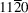 } facets is smaller (Volkov *et al.*, 2022 ▸).

Turning now to sample D, one can see in Fig. 6 ▸ that the curves at the sample orientations ψ = 15 and 30° are swapped in comparison with sample B: the intensity at the intermediate orientation ψ = 15° (red triangles) is a minimum, and not intermediate as it is for sample B. A good agreement between the experimental curves and the Monte Carlo modelling of the intensity curves for sample D is reached only with an even broader variation in the facet ratio [Fig. 7 ▸(*b*)]. The double-humped distribution of the facet ratio for this sample seems to be an artefact of the modelling of the cross sections by first producing hexagons and then cutting the corners to obtain dodecagons. The NW radii for sample D are the largest in the series [see Fig. 7 ▸(*a*)]. Comparing the average radii and the widths of their distributions in Table 1 ▸, one can see that the relative widths of the distributions (the ratios of the standard deviation to the mean value) for samples B–D are close.

Comparing now the intensity curves for sample A in Fig. 6 ▸ with those for samples B–D, one can see that the difference between the curves for different sample orientations ψ is notably weaker for sample A and the product 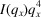 decreases at large *q*_*x*_ significantly faster. A weak orientation dependence implies roundish NW shapes and such shapes are obtained in the Monte Carlo modelling [Fig. 6 ▸(*b*)]. A fast intensity decay is a consequence of a large roughness of the side facets. The modelling gives a roughness σ = 2.3 nm, notably larger than those for samples B–D (see Table 1 ▸).

## 4. Discussion

GaN NWs grown on 3.4 µm thick Ti films sputtered on Al_2_O_3_(0001) possess remarkably small misorientation ranges, less than 2° out of plane (tilt) and less than 1° in plane (twist) (Table 1 ▸). For comparison, GaN NWs grown on the most common substrate Si(111) exhibit 3–5° tilt and twist (Jenichen *et al.*, 2011 ▸; Geelhaar *et al.*, 2011 ▸; Kaganer *et al.*, 2021 ▸) because of the formation of an amorphous SiN_*x*_ film, just a few nanometres thick, on the Si surface. The other extreme is the epitaxial growth of GaN NWs on AlN/6H-SiC 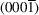 with a tilt and twist of 0.4 and 0.6°, respectively (Fernández-Garrido *et al.*, 2014 ▸).

The XRD reciprocal space-maps in Figs. 2 ▸(*a*) and 2 ▸(*c*) show that, as a result of interfacial reactions between Ti and Al_2_O_3_, the sputtered homogeneous Ti film transforms into a heterogeneous alloy containing topotaxial crystallites of Ti_3_Al and Ti_3_O. These crystals possess hexagonal symmetry, (0001) orientation, and lattice parameters intermediate between those of Al_2_O_3_ and GaN. They are topotaxially oriented with respect to the substrate with a misorientation of less than 1°. Simultaneously with the reaction of Ti with the Al_2_O_3_ substrate, the top 40–80 nm of the Ti film are converted to cubic TiN, on which the NW growth takes place (Calabrese *et al.*, 2019 ▸).

The GISAXS measurements and their Monte Carlo modelling allow us to determine the distributions of the NW radii and their cross-sectional shapes, as well as the roughness of their side facets. NWs of sample A, grown on a 1.3 µm thick Ti film, possess roundish cross-sectional shapes and a relatively high roughness of the side facets. Sample A also exhibits blue-shifted and broadened photoluminescence spectra compared with reference GaN NWs, which was attributed to the incorporation of O atoms (diffusing from the Al_2_O_3_ substrate at the employed GaN growth temperature), resulting in a background doping high enough to screen excitons and induce bandgap renormalization and band filling (Calabrese *et al.*, 2019 ▸). We thus speculate that the presence of O adatoms at the NW sidewalls may modify their surface energy, giving rise to the presence of both { 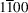 } and { 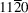 } facets. To reduce this interdiffusion, further samples were grown on 3.4 µm thick Ti films (Calabrese *et al.*, 2019 ▸).

We find that the roughness of the side NW facets of samples B–D is at least half that of sample A. However, the XRD measurements do not show a notable difference in the reciprocal-space maps between the samples. The XRD peaks from Ti_3_O remain the most intense ones, both in the symmetric reflection in Fig. 2 ▸(*a*) and in the grazing-incidence reflection in Fig. 2 ▸(*c*). We note that the latter measurement reveals the structure of the top part of the layer, due to small incidence and exit angles close to the critical angle of total external reflection. Hence, O still diffuses through the whole Ti layer despite its increased thickness. Regarding the NW shape, only sample B (even when grown at the highest temperature) exhibits purely hexagonal NW cross sections, while samples C and D are again characterized by the coexistence of { 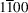 } and { 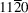 } facets. Furthermore, samples B–D display narrow excitonic transitions in their photoluminescence spectra (Calabrese *et al.*, 2020 ▸), ruling out the incorporation of O exceeding a concentration of at most 10^17^ cm^−3^. Our speculation above that O adatoms at the NW sidewalls may modify their surface energy is clearly not supported by these experimental facts.

The dodecagon cross-sectional shape of GaN NWs with both { 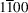 } and { 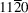 } facets has been observed in several studies. Gačević *et al.* (2015 ▸) performed selective-area MBE growth of NWs and observed a variation in the cross-sectional shape with growth time. The dodecagon shape was observed as an intermediate shape, while the final shape for a long growth time was hexagonal with { 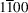 } facets. Ciechanowicz *et al.* (2021 ▸) obtained GaN NWs with a dodecagon shape in a VLS growth in Ga-rich conditions with As as antisurfactant. Pantle *et al.* (2022 ▸) found that, in selective-area MBE growth of GaN NWs, the cross-sectional faceting changed on increasing the N flux and NW diameter, from { 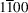 } to { 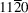 } planes. They proposed a mechanism for the shape transformation: when the Ga flux is insufficient for growth with { 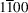 } side facets, an intermediate star-like shape is formed and then filled by reaching { 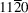 } planes.

Volkov *et al.* (2022 ▸) observed, in the self-induced MBE growth of GaN NWs on Si, hexagonal cross sections with { 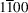 } side facets in the top parts of bundled GaN NWs on an Si substrate, and roundish shapes with both { 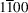 } and { 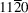 } side facets present in the bottom parts. This behaviour was considered as a transformation to the equilibrium crystal shape when the side NW surface is shadowed from the impinging fluxes, while the hexagonal shape with { 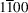 } facets was attributed to the equilibrium growth shape. A high-resolution scanning transmission microscopy study of the same samples as in the present work (Volkov & Borgardt, 2022 ▸) revealed the presence of both { 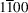 } and { 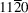 } side facets in the NWs of samples A, C and D. The NWs in sample B possess hexagonal cross sections, in agreement with Fig. 6 ▸. The distributions of the fraction of { 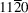 } facets in samples C and D are close to the distributions in Fig. 7 ▸(*b*), while the radii are slightly larger than those in Fig. 7 ▸(*a*).

A different possibility for the shape transformation arises from the incorporation of substantial amounts of Ga prior to GaN growth into the Ti layer (Calabrese *et al.*, 2019 ▸). After growth and during cooling, some of the Ga may be released from the Ti layer due to its reduced solubility at lower temperatures. This Ga wets the GaN NWs and turns into GaO_*x*_ upon air exposure. We note that the GISAXS intensity depends only on the density of the material and not on its crystallinity. Since crystalline GaN and amorphous GaO_*x*_ have close densities, the cross-sectional shapes obtained in Fig. 6 ▸(*b*) are those of the NWs covered with a GaO_*x*_ shell, if the latter is present. The roughness obtained from the GISAXS study also applies to the outer NW surface. The roundish shape and the coexistence of { 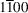 } and { 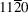 } facets would thus be characteristics of the GaO_*x*_ shell, while the GaN core may very well have a regular hexagonal shape. Plan-view transmission electron microscopy and electron-dispersive X-ray spectroscopy could be used to refute or confirm this hypothesis.

## 5. Summary

The diffusion of Al and O from the Al_2_O_3_ substrate to the sputtered Ti film gives rise to topotaxial crystallites of Ti, Ti_3_Al and Ti_3_O possessing very little misorientation with respect to the substrate. GaN NWs grown on this film are epitaxially oriented with respect to the substrate notably better than GaN NWs on Si(111).

The GISAXS intensity together with its Monte Carlo modelling are capable of providing detailed information on the NW arrays, particularly the distributions of the cross-sectional sizes of the NWs, the fractions of the { 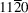 } and { 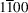 } side facets and the roughnesses of these facets. The NW radii obtained from GISAXS are systematically smaller by 2 to 5 nm than those obtained from SE micrographs. The fraction of { 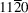 } facets is notably larger than for GaN NWs on Si(111), so that the NWs have a roundish cross-sectional shape. An exception is the sample grown at the highest temperature.

The GISAXS intensity is highly sensitive to the roughness of the side facets, a parameter not easily accessible by any other method. We found that the roughnesses of the micrometre-long side facets do not exceed a height of two or three atomic steps.

We propose that both the roughness and the shape are the result of the presence of Ga adatoms at the NW sidewall after growth and the formation of a GaO_*x*_ shell upon exposure of the NW to the ambient atmosphere.

## Figures and Tables

**Table 1:** Lengths and average radii of NWs estimated from scanning electron microscopy (SEM) (Calabrese *et al.*, 2020 ▸) and the results of the present XRD and GISAXS study The XRD measurements of FWHMs of the tilt and twist distributions were performed for the 0002 and 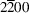 reflections, respectively. In the Monte Carlo simulation of GISAXS intensity, the FWHMs of the tilt were obtained from the *q*_*z*_ scans in Fig. 5, while the roughness of the side facets, the NW radius and the fraction 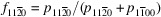 of the { 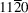 } facets were obtained from the modelling of the *I*(*q*_*x*_) intensity curves in Fig. 6. The mean values and standard deviations of the distributions for the radius and facet ratios are given.

					GISAXS (Monte Carlo)					
	SEM		XRD				Radius		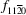	
Sample	Length (µm)	Radius (nm)	Tilt (°)	Twist (°)	Tilt (°)	Roughness r.m.s. (nm)	Mean (nm)	Width (nm)	Mean	Width
A	1.6	22	1.86	0.73	2.3	2.3	20.7	8.6	0.4	0.09
B	1.0	15	1.78	0.99	1.7	0.7	11.2	5.2	0	
C	0.7	22	1.52	0.89	1.7	1.3	17.4	8.6	0.33	0.14
D	1.2	29	1.52	0.82	2.0	0.9	24.4	10.3	0.37	0.14
